# Comparative Action of Strychnia and Veratria

**Published:** 1848-06

**Authors:** 


*Comparative Action of Strychnia and Veratria.*—Dr. Gebhort, of Moscow, has been engaged in a series of observations on the effects of veratrine as compared with those of strychnine. We shall confine our notice chiefly to the results of the author with reference to veratria, as that is an alkaloid with the properties of which we are less familiar than with those of strychnia. First, of the physiological action of veratria. Given in one-fourth or one-eighth of a grain doses to animals, it produces restlessness, difficulty of respiration, with nausea; feebleness, and intermittence of the pulse; then vomiting, borborygmi, and diarrhœa, of viscid mucus, tinged with blood; lastly, strong contractions of the abdominal muscles, tremors, and convulsions. If the dose be augmented to three or six grains, it is followed by an excessively exalted state of the nervous system, convulsions, tetanus, vomiting, frequent purging; then extreme feebleness of the muscular system, attended with tremors, and a profound nervous depression, ending in death.

Veratria applied to mucous membranes causes violent pain, inflammation, and an abundant muco-sanguineous secretion. It acts in like manner when applied to a wound. When injected into a vein, or a serous cavity, the animal becomes restless immediately, its respiration is rendered difficult, and it utters a continued cry; then follow vomiting, convulsions, diarrhœa, repeated evacuations of urine, and death.

In the human subject, the internal use of veratria occasions peculiar sensations in the stomach, alternate feelings of heat and cold, and a pricking of the skin. If the dose be increased, the stomach is tormented by a feeling of heat and burning; there are salivation, nausea, vomiting, and diarrhœa; sometimes, indeed, abundant urinary secretion and copious sweats; at other times, dull or lancinating pain in the back, in different muscular parts, and in the articulations; pains approaching in character very much to those resulting from electric shocks. Veratria determines neither a febrile condition, nor venous turgescence, nor disorder about the brain or organs of sense.

Externally it may be used in man for several months, without any other inconvenience than a sense of burning and pricking; these feelings starting from the point where it is rubbed in, and extending over the whole skin. At the same time its effects are manifested in the nerves, like those of electricity, and there is the sensation of a stream of hot water flowing along the vertebral column. The skin is rarely inflamed; but if this be delicate, or the drug be rubbed in in too great a quantity, redness of skin, with acute pain, may result, as also shocks or jerkings of the limbs, or a kind of convulsions, setting out from the part to which the veratria has been applied.

Considered therapeutically, M. Gebhort gives, as indications for its use, the existence of pain, spasm, or effusions, and of paralysis, when the last is due to effusion, or to an exhaustion of the nerves; as contra-indications, increased activity of the circulation, as by fever, inflammation, or gastro-intestinal irritation, or other disorder of the digestive organs. In these indications and contra-indications of the author are nothing very clear and concise, but rather general speculations.

M. Gebhort has tested the use of veratria in rheumatism, neuralgia, in spasmodic affections of the chest, in dropsy, and in paralysis. He has not used it in rheumatism, except after the abatement of fever and gastric disturbance. In all cases its application has been external, and of sixty cases, two only failed to be cured. The forms of neuralgia in which he has used it are those uncomplicated by organic disease, and for the most part of rheumatic origin. Of nine cases, but three remained little benefited. In forms of paralysis from cold, &c., as well as from apoplectic effusion, M. Gebhort has administered veratria with advantage. In dropsies he thinks it of great service, especially when these are unattended by organic disease, but several of the cases of dropsy given were certainly complicated with organic lesion.

He gives the alkaloid in one sixteenth of a grain doses, in the form of pills, twice a day. The quantity taken may be gradually increased to four pills per day. For external application, he unites the veratria with lard, in the proportion of five or twenty grains to an ounce of lard. Before incorporating the two, it is well to dissolve the alkaloid in a little alcohol.

Of the differential characters of strychnia and veratria the author states, that strychnine is absorbed with great rapidity, penetrates into the blood, and alters its composition; that veratrine is not absorbed; for not only, when introduced into a wound, does it not cause severe symptoms, as when injected into the veins, but the venous blood of the part into which the poison is injected produces no mischief when introduced into the veins of another animal. The contrary is the case with respect to strychnia. Strychnia has no efficacy in neuralgia, in spasmodic or in convulsive maladies; veratria, on the contrary, possesses an incontestable power over them. Strychnia has an influence over paralysis of nerves, but it causes congestion and irritation of the brain: lastly, if it have any tonic properties, by which it may be of utility in diarrhœa, dysentery, and even cholera, these properties are much impaired by the inconvenience resulting from its irritant nature, and its influence over the nervous system.

We feel very disinclined to tender our concurrence with M. Gebhort when he says that veratria is not absorbed. We think that absorption is certainly indicated by the symptoms he describes as occurring by its endemic application; and if so, we should look for the evidence of absorption still more when the veratria is introduced into an open wound. We believe, therefore, that with regard to this point there must have been some error in our author's experiments.—*Ibid.*

